# How Will In-Person and Online Grocery Shopping and Meal Consumption Activities Evolve After COVID-19?

**DOI:** 10.1177/03611981221119183

**Published:** 2022-08-26

**Authors:** Md Shahadat Hossain, Mahmudur Rahman Fatmi, Corrie Elizabeth Thirkell

**Affiliations:** 1 Civil Engineering, School of Engineering, University of British Columbia, Kelowna, British Columbia, Canada

**Keywords:** COVID-19, Eat-out, grocery shopping, online order, in-store purchase, complementarity, substitution, error correlation, joint modeling

## Abstract

COVID-19 has drastically altered the daily lives of many people, forcing them to spend more time at home. This shift significantly increased online grocery shopping and ordering for food while restrictions and social distancing measures were in place. As re-opening begins, little is known about the way virtual and in-person shopping/eating activities will evolve after the pandemic. This study adopts a multivariate ordered probit model to investigate individuals’ preferences toward the following activities after the pandemic: online grocery shopping, in-store grocery shopping, online ordering of food, and eating-out at restaurants. The model retained statistically significant error correlations among the activities, confirming the need for joint modeling. Model results suggested that individuals with lower income and with children are likely to perform grocery shopping and eating-out activities in person. Individuals owning a vehicle and a driver’s license have a higher likelihood of less frequent online shopping and more frequent in-store grocery shopping. Individuals with transit passes prefer to order groceries online and engage in eat-out activities frequently. Individuals residing in mixed land use areas prefer frequent in-store grocery shopping whereas suburban dwellers prefer it less frequently. The model confirms complementarity and substitution effects. For instance, online food ordering revealed a complementary effect on eating-out activities whereas online grocery shopping confirmed a substitution effect on in-store grocery shopping. These findings provide important behavioral insights into travel activity patterns in the post-pandemic era, which will help in understanding the inter-relationships between online and in-person shopping/eating activities, and accommodating such inter-dependencies within the travel demand forecasting models for effective policy-making.

## Introduction

In response to COVID-19, governments imposed social distancing measures, business closures, and travel restriction regulations that required people to stay at home. During the pandemic, online shopping particularly for groceries and online ordering of prepared food emerged as popular alternatives to in-person store visits and eating-out activities (*1*). For example, a study conducted in Chicago in early 2020 observed a growth of 65% for online grocery shopping and 31% growth for online food ordering (*2*). Such virtual activities have the potential to alter the way shopping and meal consumption is done on a large scale and to yield a multitude of benefits, including reductions in congestion and travel-related emissions (*3*, *4*). Many studies have looked at the trends compared with how online shopping was conducted before COVID-19 (*5*[Bibr bibr6-03611981221119183]–*7*). For example, younger, more educated, and higher-income individuals were found to be more likely to shop for food and goods online before the pandemic (*5*, *8*). Interestingly, online shopping was found to be complementary to in-store shopping rather than a substitution, and in some cases may actually generate more trips than traditional store visits, with the highest complementary relationship being for retail items (*6*). Past studies mainly focused on shopping for non-grocery items, largely because of the unavailability of online grocery and food ordering services. However, during the pandemic, online grocery shopping and food ordering, and delivery services expanded significantly, with grocery stores and food delivery apps increasing their delivery fleet sizes to accommodate the demand (*9*). This uptake in supply was triggered by the increased demand, as people were largely forced to replace their travel activities with virtual shopping and food ordering. Some studies have examined online shopping behavior during the pandemic (*2*, *8*). For example, Shamshiripour et al. (*2*) conducted a survey of Chicago residents in early 2020 and found that 13% of all respondents shopped online for groceries for the first time after restrictions had been imposed. During the pandemic, many individuals traveled significantly less which could lead to lasting changes in the way daily travel is conducted, including routine shopping trips (*7*). However, little effort has been invested in exploring the longer-term impacts of COVID-19 on food-related travel and how shopping activities might continue after the pandemic. Such understanding is critical to predict post-COVID-19 travel activity patterns and choices, and consequent vehicular emissions.

This paper investigates how in-person and online shopping and meal consumption activities are likely to evolve after the COVID-19 pandemic. Particularly, individuals’ preferences toward eating-out at restaurants and online ordering of food, and in-store and online grocery shopping, are explored. This study uses data from a web-based survey conducted from November 23, 2020 to January 9, 2021 in the Central Okanagan region of British Columbia, Canada. This survey was conducted months after the initial restrictions were instated in Central Okanagan, allowing individuals time to adjust to the new normal and for longer-term changes in behavior to emerge or for them to revert to old routines. A joint multivariate ordered probit (MVOP) modeling technique is utilized to accommodate correlations among the unobserved factors of online or in-store shopping preferences for the same activity such as grocery shopping, as well as across activities, such as grocery shopping and meal consumption. This study also examines the complementary and substitution effects between these online and in-person activities. The study extensively examines the effects of socio-demographics, access to travel mode, and built environment attributes.

The world has been in this pandemic for almost two years, at the time of writing, and it is far from over as newer variants emerge. During this period, in the Central Okanagan region of Canada, people have experienced strict travel restrictions from March 2020 to the time of writing, when most of the restrictions have been lifted, such as no group limits for indoor and outdoor dining at restaurants; however, masks are required in all public indoor settings. As we progress toward the next phase of lifting the restrictions, government agencies need to have sufficient understanding to prepare themselves to address the likely challenges, such as the need to update their travel demand models incorporating the effects of online shopping and food ordering, as well as to capitalize on opportunities such as promoting online shopping as a strategy to reduce travel-related greenhouse gas emissions. In this context, this study attempts to offer insights using the data that was collected approximately eight months after initial COVID-19 restrictions were put into place, which might have provided individuals with time to adapt to the new normal, experience, and assess the advantages and disadvantages of online and in-store shopping and meal consumption activities, and therefore be able to make an informed assessment about their preferences after the pandemic.

## Literature Review

The COVID-19 pandemic has altered the lives of many people worldwide. Governments have issued restrictions for long-distance and daily travel (*3*, *4*), resulting in many people telecommuting and online shopping for the first time (*1*). The changes in daily routine may outlast the pandemic and therefore require investigation. Online shopping for groceries and ordering prepared food are important aspects of virtual activities, as significant uptake of these online activities has been observed during the pandemic (*2*). For example, Shamshiripour et al. (*2*) conducted a survey in Chicago, U.S.A., from April to June 2020 to collect data on how travel and virtual activities had changed during COVID-19 compared with the pre-pandemic period. Descriptive analysis of the survey data revealed that approximately 20% of the survey respondents had shopped online for groceries and 40% had ordered food online from restaurants before the pandemic. After March 2020, these numbers climbed to 33% for online grocery shopping and 55% for online ordering of food from restaurants, indicating growth of 65% and 31%, respectively (*2*). These increases could result in the formation of new habits, altering the way food is purchased in the future. Conway et al. (*1*) used data from the same survey conducted during early 2020 in Chicago, and used descriptive analysis to find that 70% of the survey respondents had ordered from restaurants in the past week while 54% said they ordered from restaurants a few times a month pre-pandemic. They found that online grocery shopping increased from 23% to nearly 33% and online shopping for retail items increased from 70% to 77%. Using data from Statistics Canada, Goddard (*10*) found that 80% of food service sales were off-premises at the end of 2020 while 86% of grocery shopping was conducted in person for the same period, indicating a larger preference for in-store grocery shopping. Goddard (*10*) also found that there was a 142% increase in online ordering from December 2019 to December 2020. While these numbers indicate an increase in online ordering, Nhamo et al. (*11*) found that restaurants were operating at less than 20% capacity in many cities, including Toronto, Canada, at the end of 2020.

Most of the investigation thus far has been focused on the initial changes caused by the strict restrictions put in place during the pandemic, but there is little research on how these changes will evolve in the future. As restrictions ease and stores re-open there will likely be another shift in online ordering of food and goods. For example, in British Columbia, retail and recreation location attendance was experiencing 20% less patronage than before the pandemic during late May 2021, while in late June 2021 the same locations were experiencing 1% more patronage than before the pandemic (*12*). Shamshiripour et al. (*2*) found that 44% of respondents planned to order prepared food online more frequently and 59% of respondents planned to shop online for groceries more frequently long after the pandemic is over than they did before the pandemic, indicating a lasting change to the way groceries and food are purchased. It is important to understand how shopping and meal consumption behaviors will evolve following the pandemic and the factors influencing these changes.

The investigation of virtual shopping and ordering activities is not a new domain for transportation researchers. For example, Kim and Wang (*13*) investigated the delivery frequency of retail, grocery, and food products. They developed simultaneous equation models using data from 2018 in New York City. They examined the relationships between different types of shopping trips and travel modes in a pairwise comparison modeling framework. They found that younger individuals were more likely to order all food and goods online, they also found that males were more likely to receive food deliveries. The same study found vehicle ownership to be negatively associated with grocery delivery while living with children increased all delivery types. Smartphone ownership, higher income, and working full-time all increased the likelihood of online grocery shopping. Keeble et al. (*5*) used adjusted log regression modeling on 2018 data from Australia, Canada, Mexico, the UK, and the U.S.A. They found that being younger, male, ethnic minority, and highly educated, and the presence of children is positively correlated with online food delivery usage. Dana et al. (*8*) conducted univariate logistic regression analyses on data from Australian adults using 2018 data. They argued that younger and highly educated respondents were more likely to use online food delivery. They also found that higher income was positively associated with online food ordering.

Several studies have investigated the inter-relationship between online and in-store shopping activities. For example, three studies in California (*6*), China (*14*), and Minneapolis (*15*) found that online retail shopping had a complementary effect on in-store shopping. Lee et al. (*6*) adopted univariate ordered response models and pairwise copula-based ordered response models and argued that online shopping was associated with higher in-store shopping rates in California and that this relationship was dependent on the type of shopping being conducted. For example, online retail shopping was found to be associated with higher in-store shopping at local stores in the central business district (CBD) than at large department stores found outside the downtown district. Zhen et al. (*14*) conducted a study in Nanjing, China using a joint ordered probit model and also found that online shopping was associated with higher in-store shopping, with the strongest correlation occurring in less-frequently purchased products such as electronics and books. However, these relationships were predominant before the pandemic and may have been altered by the drastic lifestyle changes caused by social distancing measures as well as the increased accessibility for online ordering of food and groceries (*9*). However, there is limited understanding on the way shopping will be done in the post-pandemic future.

The contribution of this study is to explore how individuals’ preferences toward virtual and in-person grocery shopping and meal consumption activities are likely to evolve after the COVID-19 pandemic. Such understanding of the longer-term changes in travel behavior induced by the COVID-19 pandemic restrictions is limited. Considering the growth in online shopping activities both for groceries and food during the pandemic, it is critical to investigate how individuals’ shopping activity might evolve after the pandemic. In addition, the study extensively explores the effects of mobility tools, such as driver’s license or transit pass, and built environment and accessibility measures on shopping activity. To accommodate error correlations among the alternatives that might affect preference for these activities, a joint MVOP modeling technique is adopted. Furthermore, this study examines the complementarity and substitution effects that might exist among different virtual and in-person activities.

Some of the questions related to performing virtual and in-person activities after COVID-19 that this paper tries to answer include: do different activity types need to be modeled jointly? How do in-person and online activities for a particular purpose such as grocery shopping complement/substitute each other? How does household composition such as the presence of children, access to different travel modes such as transit pass ownership and car ownership, and built environment attributes, such as land use mix, influence in-person and virtual activities?

## Data

This study uses data from a web-based survey conducted for the Central Okanagan (includes Kelowna, West Kelowna, Vernon, Lake Country, and Peachland) in British Columbia, Canada. The survey was conducted from November 2020 to January 2021. The survey comprised the following sections: socio-demographics, work/school-related information, shopping trips and online purchases, daily travel, and residential location and vehicle ownership. The survey collected information about four time periods: before the pandemic (January 2019 to February 2020), March 2020, the week before the survey, and after the pandemic (a hypothetical futuristic scenario when the pandemic will be completely over and all restrictions will be lifted). A holiday period may have affected the frequency of trips reported for the week before the survey. However, the timing of the survey should not have much impact on the behavior during the other three periods, which are: before the pandemic, March 2020, and after the pandemic. Given that the focus of this study is analyzing the behavior in a futuristic scenario when the pandemic is completely over, the timing of the survey during the holiday season should have a limited impact.

One component of the survey was about shopping trips and online purchases, which asked respondents how frequently they shopped in-store and online for different activity types. The activities can be aggregated into the following categories: *groceries*, which includes groceries and medical supplies; *food*, which includes food prepared at restaurants; and *others*, which includes retail and other goods. The post-pandemic section was a stated preference component, which included the following options: *everyday*, *few times a week*, *few times a month*, *few times a year*, or *never.* The same options were given for the before COVID-19 and March 2020 periods. Respondents were also asked how frequently they had shopped online and in-store in the past seven days, with the options from one day to five or more days, or not at all. The socio-demographics component included data on home and work locations, individual-level attributes such as age and gender, and household-level attributes such as income and dwelling type. In addition, the study utilizes some secondary data resources. For example, the land use information is collected from the open data source of Central Okanagan. The locations of different points of interest (e.g., school, restaurants, food store, etc.) are collected from the Enhanced Point of Interest (EPOI) data.

The survey data was compared with the Census Canada data from 2016 for the Central Okanagan region of British Columbia. During this validation process, an iterative proportional fitting was adopted (*16*). The final weighted sample size is 226, and 196 of these responses contained all required data for the model. Gender and occupation were used as the calibration variables while income, education, dwelling type, household size, dwelling ownership, and age were used as validation variables. The data adequately represents the region with 77% of the variables falling within 5% of the Census distributions. For example, gender was 1% away from the census. The survey results slightly overrepresent highly educated and high-income individuals while underrepresenting individuals with less education.

**Figure 1.**
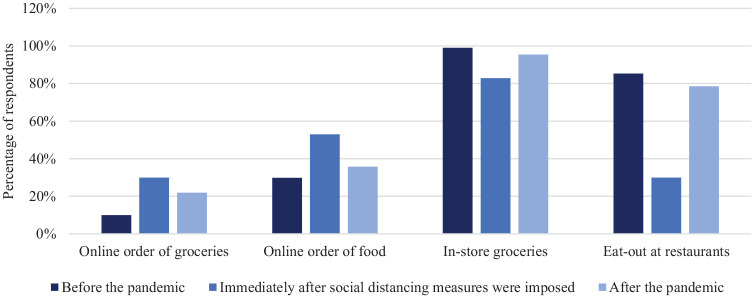
Distribution of in-person and online ordering of groceries and foods of the respondents who shopped at least a few times a month.

The COVID-19 pandemic impacted the operation of many stores worldwide and within Central Okanagan, altering hours, restricting eating-out at restaurants, and shutting non-essential businesses (*17*). In March 2020 British Columbia was experiencing significant lockdowns and newly imposed social distancing measures; these restrictions eased and residents became more comfortable with the restrictions by late 2020 (*8*, *9*). Figure 1 shows the changes for in-store and online grocery shopping, and eat-out and online ordering of food over the course of the pandemic based on the percentage of respondents conducting these activities at least a few times a month. In Figure 1, “before the pandemic” refers to before March 2020; “immediately after social distancing measures are imposed” refers to March 2020, and “after the pandemic” refers to plans for the future after all social distancing measures are lifted. The number of respondents who participated in those shopping activities in the above-mentioned timeframes are 211, 204, and 196, respectively. In March 2020, respondents who utilized online ordering of groceries increased from 10% to 30% and online ordering of food from restaurants increased from 30% to 53%. In contrast, in-store shopping for groceries decreased from 98% to 83%, and eat-out at restaurants decreased from 85% to 30%. While Figure 1 shows a clear plan to increase in-store shopping and decrease online shopping after the pandemic is over, it also shows that respondents plan to shop online more and shop in-store less than they did before the pandemic.

**Figure 2.**
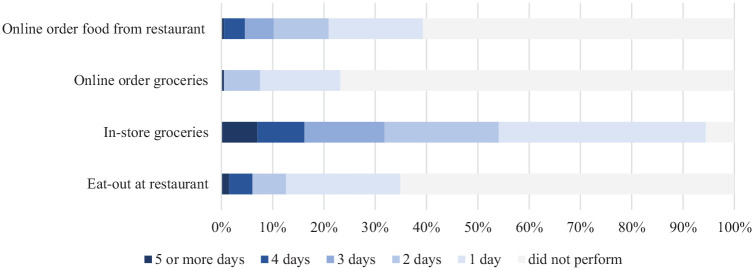
Percentage of respondents conducting in-store shopping and online ordering in the week before taking the survey.

Considering the correlation between the online ordering of multiple types of goods, 100% of respondents planning to order groceries at least a few times a month also plan to order food from restaurants at the same frequency. On the other hand, among those who are planning to online order food from restaurants a few times a month, 44% also plan to order groceries online. This indicates that respondents planning to order one type of good online are often making other types of online orders as well. In addition, respondents were asked how many days out of the last week they had conducted in-person and virtual activities for grocery shopping and food prepared at restaurants. The distribution is displayed in Figure 2. The largest participation occurred for in-store shopping for groceries, with 94% of respondents grocery shopping in-store at least once in the past week. In contrast, just 24% of respondents ordered groceries online at least once in the past week. The number of people ordering these products online is however, higher than the baseline of 10% who ordered groceries a few times a month before the pandemic. This survey was conducted months after the initial restrictions were instated in Central Okanagan, allowing time for respondents to revert to old routines, which does not seem to be the case for many of the online options. For example, 39% of respondents ordered food online from restaurants at least once in the previous week. The most frequent in-store shopping was for groceries, indicating that this remains a primary avenue for purchases.

## Modeling Approach

The study uses an MVOP modeling method to investigate the frequency of in-person and online purchases of groceries and food. The model deals with four dependent variables in a joint modeling framework that includes in-store groceries, eat-out at restaurants, online groceries, and online food purchases. The purchase frequency is coded in the following ordinal scale: 1 (never/ a few times a year), 2 (a few times a month), and 3 (a few times a week/every day). In this study context, the MVOP model comprises four independent univariate ordered probit models. The latent propensity of in-person and online shopping for groceries and food in purchase frequency can be represented as follows:

(1) $$y_{j}^{*}=\beta_{j}x_{j}+\varepsilon_{j}$$

where $y_{j}^{*}$ is the latent and continuous propensity, $x_{j}$ is the vector of independent variables, $\beta_{j}$ is the corresponding coefficient vectors, and $\varepsilon_{i}$ is the unobserved error term which is independently and identically distributed among the individuals. The observed counterpart of the latent propensity function is as follows:

(2) $$y_{j}=\left\{ \begin{array}{c} 1,\mathrm{if}\mu_{0}<y_{i}^{*}\leq \mu_{1}\\ \begin{array}{c} 2,\mathrm{if}\mu_{1}<y_{i}^{*}\leq \mu_{2}\\ \vdots \end{array}\\ k,\mathrm{if}\mu_{k-1}<y_{i}^{*}\leq \mu_{k} \end{array}\right.$$

where $\mu_{k}$ is the threshold parameter ( k = 1, 2, ….., K) and K is the highest frequency level of in-store and online activities. Since the MVOP model accommodates four dependent variables, the latent propensity can be represented as follows:

(3) $$\begin{array}{c} y_{i,1}^{*}=\beta_{1}x_{i,1}+\varepsilon_{i,1}\\ \begin{array}{c} y_{i,2}^{*}=\beta_{2}x_{i,2}+\varepsilon_{i,2}\\ \vdots \end{array}\\ y_{i,J}^{*}=\beta_{j}x_{i,J}+\varepsilon_{i,J} \end{array}$$

The error terms in the above equation are correlated and follow a standard multivariate normal distribution (*18*, *19*). The covariance matrix of the error term is as follows:

(4) $$\left(\begin{array}{c} \varepsilon_{i,1}\\ \begin{array}{c} \varepsilon_{i,2}\\ \begin{array}{c} \vdots \\ \varepsilon_{i,J} \end{array} \end{array} \end{array}\right)\sim N\left[\left(\begin{array}{c} 0\\ \begin{array}{c} 0\\ \vdots \\ 0 \end{array} \end{array}\right),\left(\begin{array}{ccc} 1 & \rho_{12} & \begin{array}{cc} \ldots & \rho_{2J} \end{array}\\ \rho_{21} & 1 & \begin{array}{cc} \rho_{22} & \rho_{2J} \end{array}\\ \begin{array}{c} \vdots \\ \rho_{J1} \end{array} & \begin{array}{c} \vdots \\ \rho_{J2} \end{array} & \begin{array}{c} \begin{array}{cc} \ddots & \vdots \end{array}\\ \begin{array}{cc} \ldots & 1 \end{array} \end{array} \end{array}\right)\right]$$

The off-diagonal elements of the above matrix represent the cross-equation error correlation that might jointly affect the frequency of in-person and online purchase activities (*14*, *20*).

This study utilized the simultaneous equation modeling technique to test the endogeneity as demonstrated in Kim and Wang (*13*). In this modeling method, the simultaneous endogenous effect can be determined as follows:

(5) $$y_{i,1}^{*}=\beta \prime_{1}y_{i,2}^{*}+\beta_{1}x_{i,1}+\varepsilon_{i,1}$$

(6) $$y_{i,2}^{*}=\beta \prime_{2}y_{i,1}^{*}+\beta_{2}x_{i,2}+\varepsilon_{i,2}$$

The following log likelihood function is maximized to estimate the model parameters.

(7) $$\mathrm{LL}=P\left(y_{i,1}=m_{i,1},y_{i,2}=m_{i,2},\cdots y_{i,J}=m_{i,J}\right)$$

The model was estimated using the “cmp” module of Stata (*21*).

## Model Results

### Goodness-of-Fit Measures

The summary statistics and the parameter estimation results of the variables retained in the final model are represented in Tables 1 and 2 respectively. The log-likelihood and the adjusted pseudo r-squared values of the MVOP model are −422.33 and 0.33 respectively. The goodness-of-fit measures were compared with the univariate ordered probit models of each activity type. This comparison reveals that the MVOP model provides a better fit. In addition, a likelihood ratio (LR) test is performed to test no correlation as the null hypothesis. The LR value is significantly higher than the critical chi-squared value in the empirical context and thus rejects the null hypothesis. Therefore, modeling the in-store and online purchases of groceries and food considering the cross-equation error correlation cannot be rejected.

**Table 1.** Summary Statistics of the Variables Used in the Multivariate Ordered Probit Model

Variables	Description	Mean percentage	Standard deviation
Socio-demographics			
Age 25–34	Individual’s age 25–34	10.20%	na
Age 35–44	Individual’s age 35–44	21.43%	na
Income <$50k	Yearly household income less than C$50,000	31.12%	na
Large household	Number of people in the household is two or more	38.78%	na
Children	Presence of children in the household	20.92%	na
Rented dwelling	Lives in a rented dwelling	30.10%	na
Single-detached	Lives in a single-detached	59.69%	na
Travel tools			
Driver’s license	Owns driver’s license	87.76%	na
Transit pass	Owns transit pass	13.78%	na
Owns vehicle and driver’s license	Owns driver’s license and a vehicle	86.22%	na
Built environment			
LUI	Land use index	0.57	0.12
CBD distance ≥3 km	Distance from residence to central business district 3 km or more	56.63%	na
Work location ≥1 km	Distance from residence to work location 1 km or more	88.78%	na
Food store distance ≥1 km	Distance from residence to the nearest food store 1 km or more	57.14%	na

*Note*: na = not applicable.

**Table 2.** Parameter Estimation Results of the Multivariate Ordered Probit Model

Variables	Grocery					Food					
	In-store		Online			Eat-out			Online		
	Coeff.	t-stat.	Coeff.		t-stat.	Coeff.	t-stat.		Coeff.		t-stat.
Socio-demographics											
Age 25–34	−0.95*	−3.03	1.11**		3.40	na	na		0.50*		1.68
Age 35–44	−0.42	−1.40	na		na	0.58	1.15		−1.62**		−4.51
Income <$50k	1.13**	3.33	na		na	1.20**	3.93		na		na
Large household	0.53**	2.31	na		na	0.47**	2.00		na		na
Children	0.51	1.48	na		na	na	na		0.92**		3.57
Rented dwelling	1.06**	2.79	na		na	na	na		na		na
Single-detached	na	na	0.56*		1.84	na	na		na		na
Travel tools											
Driver’s license	na	na	na		na	0.70	1.52		na		na
Transit pass	na	na	1.05**		2.47	1.70**	4.58		na		na
Owns vehicle and driver’s license	1.51**	3.66	−0.82*		−1.70	na	na		−1.26**		−3.85
Built environment and accessibility measures											
LUI	1.96**	2.59	na		na	na	na		na		na
CBD distance ≥3 km	−0.37*	−1.66	na		na	na	na		na		na
Work location ≥1 km	na	na	na		na	1.31**	3.58		na		na
Food store distance ≥1 km	na	na	na		na	0.49**	2.31		na		na
Endogenous variables											
Online grocery orders	−0.63**	−2.18	na		na	na	na		na		na
Online food orders	na	na	na		na	0.85*	1.85		na		na
Eat-out	na	na	na		na	na	na		0.52**		2.48
Threshold parameters											
Threshold 1	0.86	1.42	1.93**	2.91		1.67**	2.47	1.43**		2.42	
Threshold 2	2.09**	3.51	2.91**	3.73		2.86**	2.98	2.56**		4.13	
Error correlation											
In-store and online grocery	−0.79**										
In-store grocery and online food	−0.33**										
In-store grocery and eat-out	0.6**										
Online grocery and online food	0.65**										
Online grocery and eat-out	−0.034**										
Online food and eat-out	0.23**										

*Note*: Coeff. = Coefficient; t-stat. = t-statistic; na = not applicable; LUI = land use index; CBD = central business district; * 90% confidence interval; ** 95% confidence interval.

Overall, a wide variety of variables are tested in the model that is thematically categorized into the following categories: socio-demographic characteristics, travel tools, built environment, and endogenous variables. The effects of different attributes on in-store and online shopping activity for groceries and food after the pandemic are discussed below.

### In-Store and Online Groceries

Among the socio-demographic characteristics, age, household income, household composition, dwelling type, and tenure type of the household are found to be the significant factors. For example, younger (aged 25–34 years) and middle-aged (aged 35–44 years) individuals have a lower propensity to perform in-store grocery shopping. In the case of online grocery shopping, the younger age group has shown a higher propensity to perform it. The inclination of young people toward technology, such as internet use, smartphone and computer use, and website browsing might be associated with such propensity for higher frequency of online shopping. Among the income groups, lower-income individuals (yearly household income less than $50,000) prefer to perform more frequent in-store grocery shopping, which is consistent with the existing literature (*19*).

Individuals living in a larger household with two or more members have a higher propensity of performing in-store grocery shopping, which is consistent with the findings of Dias et al. (*19*). Interestingly, the presence of children in the household increases the propensity for in-store grocery shopping. Households with children are likely to have more needs and consequently might require more frequent grocery purchases. Besides, in-store grocery shopping might also serve as an out-of-home activity for households having children. Furthermore, individuals living in a rented dwelling have a higher propensity for performing in-store grocery shopping. However, individuals residing in single-detached dwellings have a higher propensity for online grocery shopping. Single-detached dwellers might be the high-income groups with higher affordability and residing in suburban areas farther from grocery stores. Their budgetary flexibility and limited multi-modal accessibility to the grocery store might lead them to prefer to order groceries online frequently.

Ownership of travel tools includes owning a driver’s license, transit pass, or household vehicle. Travel tool ownership indicates access to different travel modes that are important predictors for travel activity patterns, which consequently affect the frequencies of in-store and online shopping (*13*, *14*). Model results suggest that individuals owning both driver’s license and vehicle have a higher propensity for participating in in-store grocery shopping, whereas they have a lower propensity to perform online grocery shopping. Ownership of a car and a driver’s license offers greater travel flexibility, which might encourage them to shop in-store for groceries more frequently. Interestingly, individuals owning a transit pass have a higher propensity to engage in online grocery shopping. Going for in-store groceries using transit might be inconvenient specifically if the accessibility of the transit is not favorable, such as the distance of transit stops from home and the frequency of transit. Besides, carrying groceries from the store to the transit stop and then to the house is an added difficulty. Therefore, online grocery shopping might be a good option for people who want to avoid those inconveniences.

In the case of the built environment attributes, land use index shows a significant positive effect for in-store grocery shopping. Individuals living in areas with a higher mix of land use diversity typically have well connected multi-modal accessibility to grocery stores which are often within a close distance of their residences (*20*). Therefore, they are likely to engage in more frequent in-store grocery shopping. Individuals living farther from the urban core (distance to CBD from the residence ≥3 km) have a lower propensity to participate in in-store grocery shopping. Making a longer trip for groceries and the time constraints of such a trip might not be a suitable option for suburban dwellers. Therefore, they might be inclined toward less frequent but bigger grocery purchases.

### Eating-Out and Online Ordering of Prepared Food

Younger individuals have a higher propensity to participate in online food ordering. Middle-aged individuals are likely to order food online less frequently, whereas they are likely to participate in eat-out activities more frequently. Lower-income households are more inclined toward eat-out activities. The presence of children in the household has a positive effect on ordering food online more frequently. Households with children are more likely to consume more food and sometimes online food ordering might be a more attractive and quick option to avoid kitchen duties. In the case of travel tool ownership, owning a driver’s license and a transit pass is likely to encourage individuals to engage in eat-out activities more frequently. On the other hand, owning both vehicle and driver’s license might lead to ordering food online less frequently.

In the case of built environment attributes, individuals living farther from their work location are likely to participate in eat-out activities more frequently. After the travel restrictions are relaxed, individuals are expected to travel to work more. Thus, they might participate in eat-out activities while going to or returning from work. In addition, they might socialize with colleagues by participating in eat-out activities more frequently. Individuals living farther from the food store have a higher propensity to dine in restaurants. These results indicate that individuals might be interested in going back to the pre-pandemic lifestyle while also practicing the habit of online food ordering that was acquired during the pandemic.

### Endogeneity Effects and Error Correlation

These joint model results reveal the complex inter-relationships between in-store and virtual activities for grocery shopping and preparing food, by testing for complementary and substitution effects. For example, results suggest that frequent online food orders have a complementary relationship with frequent eat-out activities. This might be a longer-term effect of COVID-19. People might have acquired the habit of online food ordering during the pandemic, while they also prefer to return to eat-out activities in the post-pandemic era. As a result, the more individuals order food online, the more they eat-out after the pandemic. Online groceries reveal a substitution effect on in-store groceries. It is expected that individuals who order groceries online are more inclined toward staying and spending more time at home and therefore less inclined toward traveling for grocery shopping.

In the case of cross-equation error correlation, all error correlations are statistically significant. A positive relationship is found between online grocery and online food ordering, which might indicate that unobserved factors that increase online grocery shopping frequency also increase online food ordering activities. A negative relationship is confirmed between in-store grocery and online grocery, in-store grocery and online food, and online grocery and eat-out. This demonstrates that the unobserved factors have an inverse effect on their shopping frequency.

### Marginal Effects

The parameter estimation results of the MVOP model represented in Table 2 do not reflect the magnitude of the impact of independent variables on online/in-store groceries and food purchasing activities. Therefore, the marginal effects of the variables on each preference level of in-person and online purchases of groceries and food are estimated and presented in Table 3. The results suggest that the presence of children in the household might increase the probability of frequent in-store grocery shopping by 12.1% compared with households without children. Individuals owning a vehicle and a driver’s license are 35.6% more likely to make frequent in-store grocery purchases than individuals without vehicles and a driver’s license. Living in a high land use mix areas has a substantial positive impact on frequent in-store grocery purchases.

**Table 3.** Marginal Effects of the Variables Retained in the Multivariate Ordered Probit Model

Variables	Grocery						Food					
	In-store			Online			Eat-out			Online		
	Never/few times a year	Few times a month	Few times a week/every day	Never/few times a year	Few times a month	Few times a week/every day	Never/few times a year	Few times a month	Few times a week/every day	Never/few times a year	Few times a month	Few times a week/every day
Socio-demographics												
Age 25–34	**0.075**	**0.149**	**−0.223**	**−0.162**	**0.088**	0.074	na	na	na	**−0.077**	**0.034**	**0.018**
Age 35–44	0.033	0.066	**−**0.099	na	na	na	**−0.309**	0.329	**0.329**	**0.174**	**−0.077**	**−0.098**
Income <$50k	**−0.089**	**−0.177**	**0.266**	na	na	na	**−0.196**	0.209	**0.209**	na	na	na
Large household	**−0.042**	**−0.083**	**0.125**	na	na	na	**−0.091**	0.098	**0.098**	na	na	na
Children	**−**0.041	**−**0.081	**0.121**	na	na	na	na	na	na	**−0.143**	**0.063**	**0.034**
Rented dwelling	**−0.084**	**−0.167**	**0.251**	na	na	na	na	na	na	na	na	na
Single-detached	na	na	na	**−0.146**	**0.080**	0.067	na	na	na	na	na	na
Travel tools												
Driver’s license	na	na	na	na	na	na	**−0.136**	0.145	**0.145**	na	na	na
Transit pass	na	na	na	**−0.277**	**0.151**	0.126	**−0.328**	0.350	**0.350**	na	na	na
Owns vehicle and driver’s license	**−0.119**	**−0.237**	**0.356**	**0.010**	**−0.006**	**−**0.005	na	na	na	**0.194**	**−0.085**	**−0.046**
Built environment												
LUI	**−0.155**	**−0.308**	**0.462**	na	na	na	na	na	na	na	na	na
CBD distance ≥3 km	0.029	**0.057**	**−0.086**	na	na	na	na	na	na	na	na	na
Work location ≥1 km	na	na	na	na	na	na	**−0.225**	0.240	**0.240**	na	na	na
Food store distance ≥1 km	na	na	na	na	na	na	**−0.095**	0.101	**0.101**	na	na	na

*Note*: na = not applicable; LUI = land use index; CBD = central business district. Coefficients in bold font are significant at least at 90% confidence interval.

## Conclusion

This paper presents findings on individuals’ preferences toward grocery and restaurant meal consumption activities after the COVID-19 pandemic. The scope of the study includes both in-person and online methods of shopping/eating and ordering for delivery at home. Data come from a web-based survey conducted in the Central Okanagan region of British Columbia, Canada, from November 2020 to January 2021. The survey collected information on travel behavior for the following time points: pre, during, and post-COVID-19. A stated preference component of the survey collected information on the in-person and online activities for groceries and food prepared at restaurants after the pandemic. This information is used to develop a joint MVOP model. This MVOP jointly models four endogenous variables: online grocery shopping, in-store grocery shopping, online ordering of food from restaurants, and eating-out at restaurants. Individuals’ preferences are coded in the following ordinal scale: rare, sometimes, and frequent. The purpose of adopting this joint modeling technique is to accommodate correlations among the error components of the alternatives and capture the true causal effects to provide insights into the complementarity and substitution impacts between virtual and in-person activities.

The model results suggest that all error covariances are statistically significant, indicating the need to model the four activities jointly. Further, model results reveal insights into how grocery shopping and eat-out activities might evolve in the post-pandemic era, in relation to socio-demographics, travel tool ownership, and built environment attributes. For example, lower-income individuals have a higher propensity to perform grocery and meal consumption activities in person. The presence of children correlates to a higher propensity for online food ordering, as well as in-store grocery shopping. Individuals with a driver’s license and a vehicle have a propensity for less frequent online ordering of both food and groceries, whereas they are likely to make frequent in-store grocery shopping trips. Individuals with a transit pass have a higher propensity for ordering groceries online, whereas they prefer to engage frequently in eating-out activities at restaurants. Individuals residing in higher mixed land use areas are likely to engage in frequent in-store grocery shopping. Individuals residing farther from the urban core are likely to prefer less frequent in-person grocery shopping. One of the key findings of this study is to confirm the complementary and substitution effects. The model confirms the complementary effect for meal consumption activities—indicating that more online food ordering is associated with more eat-out activities. A substitution effect is confirmed for grocery shopping, which suggests that more online grocery shopping is associated with less in-store grocery shopping.

This study has certain limitations. First, the study used a small sample size. The sample constraints mean that the effects of some of the important attributes, such as variables representing built environment and travel characteristics, were not found to be significant for all alternatives. Although those variables were tested for all the choice alternatives, they yielded less significant parameters or counterintuitive results and therefore were removed from the final model. Second, the limited data resources meant that the effects of attitudes could not be tested in this study. Such modeling with a small sample size is challenging and might affect the parameter estimation results. However, the statistically significant results clearly dictate the behavioral changes in the post-pandemic period. To better understand the impact of sample size and demographics of the study area, future studies should focus on more comprehensive data collection for modeling purposes. Besides, further studies should investigate the impact of the characteristics of different geographical locations on the behavioral changes in the post-pandemic period by developing and comparing the model results for those locations. Third, the study utilized a web-based survey for data collection. In this regard, there might be some biases in the responses assuming that the participants of the survey might be more technology savvy and more competent in using smartphones, computers, and web browsing. Therefore, they might be more inclined toward online ordering of food and groceries. It should also be noted that the survey was conducted over a major shopping time for Canadians, which could affect the number of shopping trips for the week before the survey. Another limitation of the study is the inability to differentiate between delivery and pickup, because of the unavailability of such information. Future research should collect information on the nature of online orders such as delivery versus pickup to investigate the variables that influence online ordering for pickup or delivery.

Overall, the study provides important behavioral insights into the longer-term effects of COVID-19 on in-store and online shopping/eating activities, which might help in understanding the activity travel pattern in the post-pandemic period, and their congestion and emissions implications. For example, the substitution effects retained for the online ordering of groceries on in-store grocery shopping indicate a likely decrease in trips, resulting in a likely reduction of vehicle kilometers traveled, congestion, and greenhouse gas emissions. On the other hand, the complementary effects of online food ordering on eat-out activities imply a likely increase in travel activities. These implications are also related to the nature of the online order, whether delivery or pickup. Delivery by the vendor to the individual’s home is likely to increase vehicular traffic on the road. Similarly, picking up orders will increase travel activities as individuals have to make a trip to go and pick up the products or food. Future research should consider the nature of the online order and invest more effort in analyzing the impacts on the road network and the environment.

The behavior during COVID-19 is evolving, and how it might be shaped in the long-term is associated with many uncertainties. Therefore, more efforts are required to continue data collection and gather information during and after the pandemic. In this context, this study will serve as a baseline for future studies to compare with individuals’ actual changes in behavior after the pandemic. Future research should focus on forecasting and scenario testing to better understand the pre- and post-pandemic behavioral changes for grocery shopping and meal consumption activities.
